# Multi-DOA estimation based on the KR image tensor and improved estimation network

**DOI:** 10.1038/s41598-021-85864-5

**Published:** 2021-03-18

**Authors:** Ye Yuan, Shuang Wu, Yong Yang, Naichang Yuan

**Affiliations:** 1 State Key Laboratory of Complex Electromagnetic Environment Elects on Electronics and Information System, National University of Defense Technology, Changsha, 410073 China; 2 Innovation Academy for Microsatellites, Chinese Academy of Sciences, Shanghai, 200120 China

**Keywords:** Electrical and electronic engineering, Information technology

## Abstract

Deep neural networks have shown great performance for direction-of-arrival (DOA) estimation problem, but it is necessary to design some suitable networks to solve the multi-DOA estimation problem. In this paper, we use Khatri–Rao product to increase the degree of freedom of antenna array and obtain the image tensor of covariance matrix, then we propose an improved estimation network to process the tensor. We use the curriculum learning scheme and partial label strategy to develop a CurriculumNet training scheme. The training/validation results shows that the proposed training scheme can increase the generalization of the estimation network and improve the accuracy of network around $10\%$. The estimation performance of the proposed network shows high-resolution results, which can distinguish two adjacent signals with angle difference of $3^\circ$. Moreover, the proposed estimation network has root mean square estimation error lower than $1^\circ$ when signal noise ratio equals $-\,10\,{\mathrm {dB}}$ and can estimate DOAs precisely by only 8 snapshots, which performs much better than prior deep neural network based estimation methods and can estimate multi-DOA results under hostile estimation environments.

## Introduction

Direction-of-arrival (DOA) estimation technologies are widely used in the modern radar and communication systems^1–5^. In the past decades, researchers have presented many super-resolution estimation methods. Two of the most classic methods (subspace based methods) are known as the multiple signal classification (MUSIC) algorithm and estimating signal parameter via rotational invariance techniques (ESPRIT) algorithm^6,7^.

Many articles have proposed numerous variants of MUSIC and ESPRIT methods^8–10^, some improvements of the estimation performance have already been made. Researchers always combine the MUSIC method with other technologies to enhance the estimation performance, such as particle swarm optimization based MUSIC (PSO-MUSIC) method used for tracking sound source^11^ and low-degree root-MUSIC method used for fast estimation^12^. For the improvements of ESPRIT method, it is important to enhance the accuracy of the method and improve the estimation speed^13,14^.

As an interesting mathematical tool, the Khatri–Rao (KR) product has been introduced into the DOA estimation field. Researchers have proposed many efficient KR product based methods, such as the KR subspace approach used for quasi-stationary signals^15^ and the KR product based MUSIC algorithm used for Gaussian signals^16^. Researchers also find that the KR product can enhance the degrees of freedom (DOF) for arrays. Based on this conception, the so-called nested array has been proposed both in the 1-D^16^ and 2-D^17,18^ estimation fields. Although these KR subspace based methods have been well developed and many varietal algorithms have been proposed^19,20^, the defects of these methods are obvious. Firstly, the estimation performance of subspace based methods relies on the prior information, and the prior information is hard to obtain in reality^21^. Secondly, the decomposition operation is computationally complex. For MUSIC based method, the spectrum searching operation also cost plenty of computing resources. Therefore, it is necessary to find some alternative methods with independence of prior information to solve the DOA estimation problem.

Prior articles have proved that DOA estimation problem is a kind of convexity problem^22,23^, whose sparsity property can be solved by the $\ell _p$-norm approximation^24,25^. Many convex optimization algorithms have been used in the DOA estimation field such as the $\ell _1$-norm to approximatively solve the $\ell _0$-norm constrained minimization problem and obtain the off-grid DOA estimation results^26^, and the low-rank matrix approximation method to recover the DOA information from the array observation matrix and a weakly convex function in LRMA^27^. Machine learning-based methods are data-driven, they do not rely on prior information about array geometries or signal forms. Researchers have brought some efficient machine learning methods into the DOA estimation field, such as radial basis function neural network (RBF-NN)^28^, support vector regression (SVR)^29^, and neural network^30^. These machine learning based methods are also used for dealing with special signal forms, such as the sparse Bayesian learning (SBL) method for coherent sources^31^ and the modified quantum genetic algorithm (QGA) for wideband signals^32^. These methods can automatically learn the probability density function (PDF) of DOA^33^, therefore, the estimation method based on machine learning need less prior knowledge from the signal^34^. But the training and estimation processes become more complex, and the accuracy of the estimating results depends on the parameters of methods. These parameters are derived by some verification experiments and they are hard to obtain^35^, therefore, we need to develop some convenient estimation methods with more ability of self-adaption and intelligent.

With the rapid development of artificial intelligent (AI) technologies^36^, some AI based estimation methods have been proposed in recent years. Researchers introduce the deep neural network (DNN) into the DOA estimation field and develop a DNN group to do the uniform linear array (ULA) estimation task^37^. Despite that the proposed method does not rely on the prior information and shows robustness with the array imperfections, it has some defects which need to be overcome. Firstly, while estimating DOA information by using the proposed DNN group, the DOAs of two adjacent receipt signals will need at least $10^{\circ }$ interval. This means that the resolution of the proposed DNN group is limited, and the accuracy of estimating results will decline if DOAs of signals are getting too closer. Secondly, the ULA used in this article contains 10 sensors, which means the array aperture is pretty large. Thirdly, these DNN based methods use the vector of array observation data or vectorized covariance matrix as the input tensor of the network^38^. This operation ignores the structure of covariance matrix and can reduce the quantity of information. In order to make full use of the information hidden in the covariance matrix, researchers also propose some deep convolution network (DCN) based methods, such as the DCN estimator used for estimating multi-DOA with sparse prior^39^, the DCN used for estimating DOAs of multi-speaker with noise signals^40^, and the de-multipath DCN models used for achieving multipath DOA estimation^41^. Although these DCN based methods use the convolutional layers to extract the deep features of covariance matrix, the pre-processing operation proposed in these articles^39–41^ can complicate the estimation problem and cause time-consuming.

This paper mainly focus on three problems of the deep neural network based DOA estimation methods: the lack of information hidden in the covariance matrix, the difficulty to train the network with massive scale of output categories related to the DOA spectrum, and the problem of multi-label classification problem (with respect with the multi-DOA estimation problem). Firstly, we use the KR product to increase the degree of freedom of antenna array and obtain the image tensor of covariance matrix. This operation can help the neural network process the covariance matrix with larger size and more information corresponding to DOAs of signals. We discuss the theory and features of the covariance matrix obtained by the KR product in the “Methods” section. Then we transform the DOA estimation problem into an image classification problem and propose an improved estimation network to finish the multi-DOA estimation task. In order to deal with the massive scale of categories, we use the curriculum learning scheme (CLS) to increase the generalization of the proposed estimation network. We also introduce the partial label strategy (PLS) into the network to obtain multi-classification results with high performance. The details of network and improved training algorithm design are also shown in the “Methods” section. We discuss the effectiveness of the proposed training algorithm and the estimating performance of proposed estimation network in the “Results” section. The character of estimation errors, the RMSE responses under different estimation environment, and the resolution of the proposed estimation network is studied. The comparison results with prior estimation algorithms show the excellent stability of the proposed estimation network. Finally, we conclude this paper in the “Discussion” section.

## Methods

### KR product and image tensor

We assume that *K* individual Gaussian signals spatially distributed at the direction of ${\varvec{\theta }} = \left[ \theta _1, \theta _2, \dots , \theta _K\right]$. All signals simultaneously impinge on an ULA with *M* sensors. The array observation data ${\varvec{x}}\left( n\right)$ can be expressed as Eq. (1):

1 $$\begin{aligned} {\varvec{x}}\left( n\right) = {\varvec{A}}{\varvec{s}}\left( n\right) + {\varvec{v}}\left( n\right) , \end{aligned}$$

where *n* denotes the *n*th snapshot. ${\varvec{s}}\left( n\right)$ (resp. ${\varvec{v}}\left( n\right)$) denotes the signal vector (resp. noise vector). ${\varvec{A}} = \left[ {\varvec{a}}\left( \theta _1\right) , {\varvec{a}}\left( \theta _2\right) , \dots , {\varvec{a}}\left( \theta _K\right) \right]$ represents the array manifold, where ${\varvec{a}}\left( \theta _k\right)$ can be expressed as Eq. (2):

2 $$\begin{aligned} {\varvec{a}}\left( \theta _k\right) = \left[ 1, e^{-{\mathrm {j}}2\pi \frac{d}{\lambda }\sin \theta _k}, \dots , e^{-{\mathrm {j}}2\pi \frac{\left( M-1\right) d}{\lambda }\sin \theta _k}\right] ^T, \end{aligned}$$

where $\lambda$ denotes the wavelength of signals. We set the inter-sensor space of ULA $d = \lambda /2$. The covariance matrix ${\varvec{R}}$ of observation data can be calculated as Eq. (3):

3 $$\begin{aligned} {\varvec{R}} = E\{{\varvec{x}}{\varvec{x}}^H\} = \frac{1}{N}\sum _{n = 1}^N{\varvec{x}}\left( n\right) {\varvec{x}}^H\left( n\right) = {\varvec{A}}{\varvec{R}}_{s}{\varvec{A}}^H + {\sigma _v}^2{\varvec{I}}_M. \end{aligned}$$

where *N* denotes the number of snapshots. ${\varvec{R}}_{s} = {\mathrm {diag}}\left( {\varvec{\sigma }}\right)$ represents the power matrix of signals (${\varvec{\sigma }}= \left[ {\sigma _1}^2, {\sigma _2}^2, \dots , {\sigma _K}^2\right] ^T$). ${\sigma _v}^2$ denotes the power of white noise and ${\varvec{I}}_M$ denotes the $M\times M$ unit matrix. The vectorized ${\varvec{R}}$ can be regarded as signals impinge on a new array whose manifold is given by $\hat{{\varvec{A}}} = {\varvec{A}}^*\odot {\varvec{A}}$^15^.

4 $$\begin{aligned} {\varvec{y}} = {\mathrm {vec}}({\varvec{R}}) = {\mathrm {vec}}\left[ \sum _{k = 1}^K{\sigma _k}^2\left( {\varvec{a}}\left( \theta _k\right) {\varvec{a}}^H\left( \theta _k\right) \right) \right] + {\sigma _v}^2 \vec {{\varvec{1}}}_M = \left( {\varvec{A}}^*\odot {\varvec{A}}\right) {\varvec{\sigma} }+ {\sigma _v}^2 \vec {{\varvec{1}}}_M, \end{aligned}$$

where $\odot$ denotes the KR product. $\vec {{\varvec{1}}}_M = \left[ {\varvec{e}}_1^T, {\varvec{e}}_2^T, \dots , {\varvec{e}}_M^T\right] ^T$ and ${\varvec{e}}_m$ denotes a column vector with *M* elements. The *m*th element of ${\varvec{e}}_m$ equals 1 while others equal 0.

The KR product can also generate several identical elements in ${\varvec{y}}$^16^, therefore, we remove the redundant elements and obtain a new vector ${\hat{{\varvec{y}}}}$. The new covariance matrix ${\hat{{\varvec{R}}}}$ can be expressed as Eq. (5):

5 $$\begin{aligned} {\hat{{\varvec{R}}}} = {\hat{{\varvec{y}}}}{\hat{{\varvec{y}}}}^H = {\hat{{\varvec{A}}}}{\varvec{\sigma }}{\varvec{\sigma} }^H{\hat{{\varvec{A}}}}^H + {\sigma _v}^4{\hat{{\varvec{e}}}}{\hat{{\varvec{e}}}}^H + {\sigma _v}^2{\hat{{\varvec{A}}}}{\varvec{\sigma }}{\hat{{\varvec{e}}}}^H + {\sigma _v}^2{\hat{{\varvec{e}}}}{\varvec{\sigma }}^H{\hat{{\varvec{A}}}}^H, \end{aligned}$$

where ${\hat{{\varvec{e}}}}$ denotes a vector whose $\left( M\left( M - 1\right) /2 + 1\right)$th element equals 1 while others equal 0.

**Figure 1:**
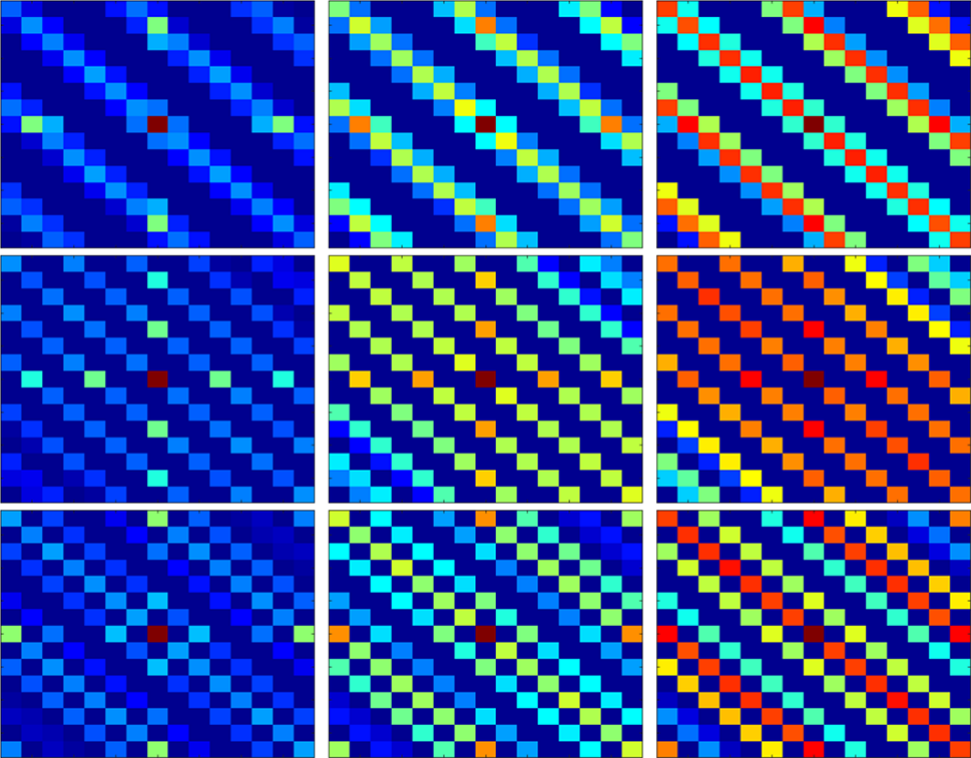
Image feature of matrix ${\mathrm {Re}}\{\hat{{\varvec{R}}}\}$. From top to bottom, $\theta = 20^\circ$, $40^\circ$, and $60^\circ$. From left to right, the SNR equals − 5 dB, 0 dB, and 5 dB.

We reshape ${\hat{{\varvec{R}}}}$ into an image tensor ${\mathscr {R}}$ where the real part matrix ${\mathrm {Re}}\{\hat{{\varvec{R}}}\}$ and imaginary part matrix ${\mathrm {Im}}\{\hat{{\varvec{R}}}\}$ are two individual channels of ${\mathscr {R}}$. We propose a method to extract DOA information from the image feature of ${\mathscr {R}}$, and the advantages of this method can be summarized as follows:

- The use of tensor ${\mathscr {R}}$ can be processed by the convolution operation, which is an efficient tool with satisfying robustness^42^.
- The KR product can extend the DOF of array, which allows the array to estimate more DOAs^17,18^. Furthermore, KR product can ensure the size of ${\mathscr {R}}$ and help the network obtain more information.
- We investigate the image feature of ${\mathscr {R}}$ (shown in Fig. 1, here we set $M = 8$) and find out that the main structure of ${\mathscr {R}}$ only corresponding to $\theta$. The estimation environment can hardly influence the image feature of ${\mathscr {R}}$. Therefore, it is possible to develop some estimation methods with high performance under harsh environment.

Based on these conceptions, we design an improved estimation network to deal with the multi-DOA estimation problem with the proposed KR image tensor ${\mathscr {R}}$.

### Curriculum learning and partial label

**Figure 2:**
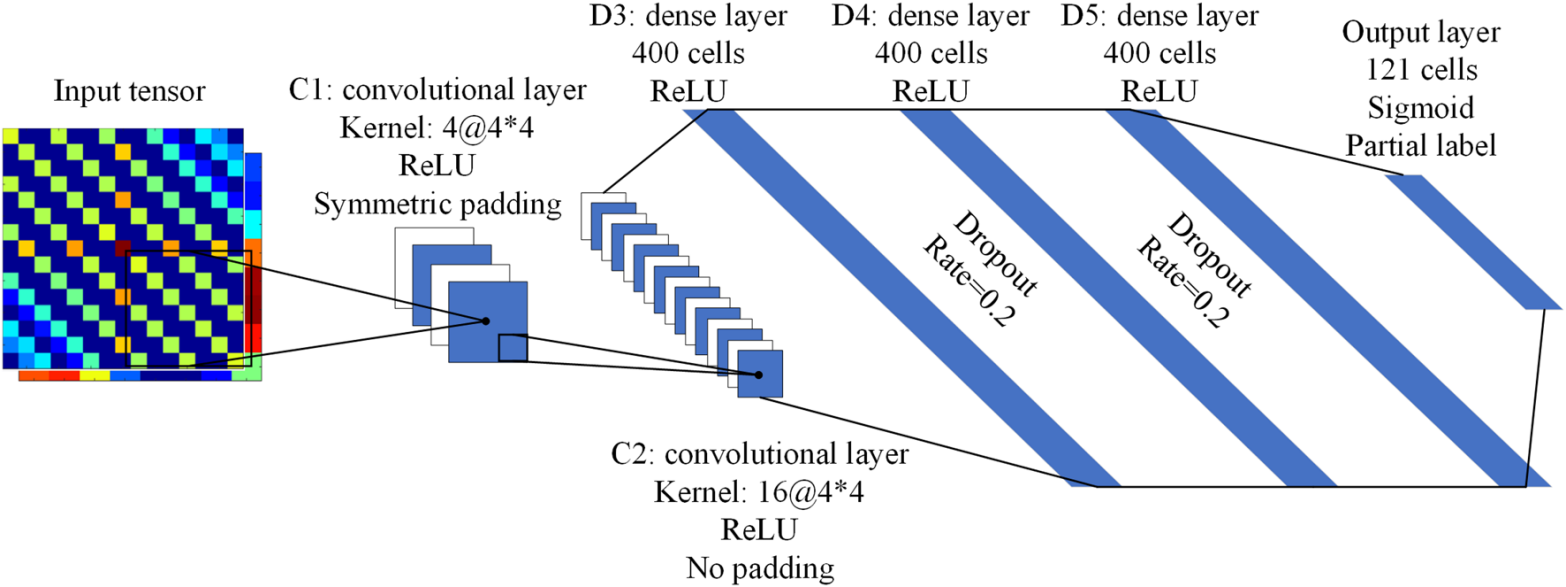
Framework of the proposed simple estimation network.

Figure 2 shows the framework of the proposed estimation network. It contains 6 layers: two convolutional layers, three dense layers, and one output layer. Two convolutional layers contain 4 and 16 individual feature maps, respectively. For the kernel size of the convolutional layers, researchers have already proved that symmetric padding can release the generalization capabilities of even-sized kernels^43^. The convolution with even-sized kernels and symmetric same padding can have outperformance than odd-sized kernels in the image classification tasks^43^. Therefore, we use the kernel with an even size as $4\times 4$ and the symmetric same padding mode in the first convolutional layer. We set the convolution step as 2.

We use three dense layers with same length of outputs to build the full connected part of the estimation network. Each dense layer contains 400 cells, which is chosen to reach the trad-off between the nonlinear expressivity (improves with larger network scale) and over-fitting risk of the network (aggravates with more network parameters). We use the dropout technology to avoid over-fitting of the proposed estimation network. As it is shown in Fig. 2, two dropout layers are added behind the first and the second dense layers. We set the dropout rate of these two layers as 0.2. For the output layer, we divide the angle space of $\theta$ (from $-60^{\circ }$ to $60^{\circ }$) by $1^{\circ }$ and use 121 output cells to represent the spectrum of $\theta$, therefore, the output layer contains 121 categories.

As it is illustrated in Fig. 2, we use the ReLU function as the activations of the first five layers, while the output layer uses the Sigmoid function as its activations in order to achieve multi-classification effect^44^. As a consequence, the proposed estimation network transforms the multi-DOA estimation problem into a multi-label classification problem of image tensor.

We introduce the CLS^45^ into the estimation network. Researchers have already shown that the CLS is a powerful tool for the weakly supervised learning^45^. With the CLS, the proposed estimation network can have better estimation performance under highly noise situation (low SNR and small number of snapshots *N*). This character also shows that the CLS can indirectly improve the generalization capability of the proposed estimation network. We also use the PLS^44^ to achieve efficient multi-label classification. PLS is related to the so-called never-ending learning (NEL) paradigm^44^, which use the previously learned knowledge to improve the learning of the network. Moreover, the PLS can predict the missing labels using the learned network. The proposed learning scheme is shown in Algorithm 1, and the training details are shown as follows.

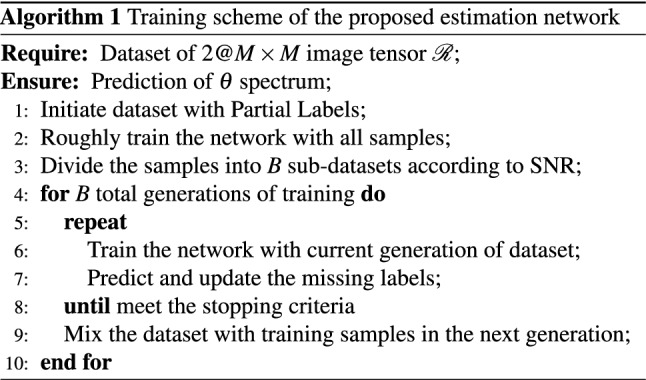



At first, we roughly train the network with all training samples and obtain a basic estimation network. Then we generate *B* clusters of samples according to the SNR information and use them as the sub-datasets. The training samples is also divided into *B* subsets and sorted from the sample subsets with higher SNR to the sample subsets with lower SNR. We denote the sorted subsets as ${\mathscr {D}}_i, i = 1, 2,\dots , B$ and the training samples in the current generation as ${\mathscr {D}}_C$. During each generation of training, we continuously mix the subsets together as the training samples: ${\mathscr {D}}_C = {\mathscr {D}}_C \cup {\mathscr {D}}_i$, where the initial ${\mathscr {D}}_C = \emptyset$.

To deal with the multi-DOA estimation problem, we also use the PLS to decrease the scale of dataset space and obtain multi-classification results with high performance.

We denote the training data as ${\mathscr {D}} = \left\{ \left( {\mathscr {R}}^{\left( 1\right) },{\varvec{z}}^{\left( 1\right) }\right) , \left( {\mathscr {R}}^{\left( 2\right) },{\varvec{z}}^{\left( 2\right) }\right) , \dots , \left( {\mathscr {R}}^{\left( S\right) },{\varvec{z}}^{\left( S\right) }\right) \right\}$, where ${\mathscr {R}}^{\left( s\right) }$ (resp. ${\varvec{z}}^{\left( s\right) }$) denotes the *s*th sample (resp. label vector). *S* denotes the number of training data. ${\varvec{z}}^{\left( s\right) } = \left[ z_1^{\left( s\right) }, z_2^{\left( s\right) }, \dots , z_{C}^{\left( s\right) }\right]$ where $C = 121$ represents the number of output categories. We denote $z_c^{\left( s\right) } = 1$ (resp. $-1$ and 0) means the present (resp. absent and unknown) of this label. We also define a hyperparameter $p\%$ to randomly choose which labels should be set as present labels.

The partial labels is collaborated with partial-BCE loss^44^. Unlike the standard BCE loss, the partial-BCE loss use a normalization probability distribution $g(\bullet )$ to give the same importance to each sample, which can adapt itself to the number of known labels. This special character gives PLS the ability to increase the generalization of the network^44^. We denote the *s*th output of estimation network as ${\varvec{w}}^{\left( s\right) } = \left[ w_1^{\left( s\right) }, w_2^{\left( s\right) }, \dots , w_C^{\left( s\right) }\right]$, and the partial-BCE loss function ${\mathscr {L}}$ can be expressed as Eq. (6):

6 $$\begin{aligned} {\mathscr {L}} = - \frac{g(p)}{CS}\sum _{s = 1}^S\sum _{c = 1}^C\left[ {\varvec{1}}_{\left[ z_c^{\left( s\right) } = 1\right] }\log \left( \frac{1}{1 + \exp (-w_c^{\left( s\right) })}\right) + {\varvec{1}}_{\left[ z_c^{\left( s\right) } = -1\right] }\log \left( \frac{-w_c^{\left( s\right) }}{1 + \exp (-w_c^{\left( s\right) })}\right) \right] , \end{aligned}$$

where ${\varvec{1}}_{\left[ \bullet \right] }$ denotes a binary detector of $z_c$. $g(\bullet )$ is the normalization probability distribution corresponding to the proportion of present labels.

7 $$\begin{aligned} g(p) = \alpha p^\gamma + \beta , \end{aligned}$$

where $\alpha$, $\beta$, and $\gamma$ are hyperparameters which control the value of *g*. We fix the value of $\gamma$ at 3 and set $g(0) = 5$, then we can obtain that $\alpha = -4$ and $\beta = 5$.

In order to recover the missing labels, we implement the Bayesian uncertainty strategy (BUS)^46^ after each generation of training. We define a score vector ${\varvec{v}}^{\left( s\right) } = \left[ v_1^{\left( s\right) }, v_2^{\left( s\right) }, \dots , v_C^{\left( s\right) }\right]$ to represent the prediction of label vector. We also define a hyperparameter $\rho$ as the detection threshold. The objective function of prediction can be expressed as Eq. (8):

8 $$\begin{aligned} \min _{{\mathscr {F}}\in {\mathscr {R}}^{16{@}3\times 3}, {\varvec{v}}\in \left\{ 0,1\right\} ^{S\times C}} J({\mathscr {F}},{\varvec{v}}) = \kappa \left\| {\mathscr {F}}\right\| ^2 + G({\varvec{v}};\rho ) + \frac{1}{SC}\sum _{s = 1}^S\sum _{c = 1}^C v_c^{\left( s\right) }{\mathscr {L}}_c\left( w_c^{\left( s\right) }, z_c^{\left( s\right) }\right) , \end{aligned}$$

where $\kappa$ is a positive value which controls the weight of $\left\| {\mathscr {F}}\right\|$. ${\mathscr {L}}_c$ denotes the loss of the *c*th category. $G(\bullet )$ denotes the curriculum learning scheme mentioned before. The label vector ${\varvec{v}}$ can be predicted as Eq. (9):

9 $$\begin{aligned} v_c^{\left( s\right) } =\infty {\left[ {\mathscr {U}}({\mathscr {R}}^{\left( s\right) })_c\le \rho \right] }, \end{aligned}$$

where ${\mathscr {U}}(\bullet )_c$ denotes the Bayesian uncertainty^46^ of the *c*th category in the *s*th sample.

The whole training scheme shown in Algorithm 1 make the proposed estimation network become an improved CurriculumNet, which is motivated by human learning^45^. Rather than regurgitating, more details of CLS and PLS are listed in prior articles^44,45^. With the proposed training scheme, the proposed estimation network can gradually learn from easier tasks (datasets with higher SNR) to harder ones (datasets with lower SNR), which is helpful to improve the generalization of the proposed network.

## Results

### Implementation details

In order to verify the reasonable design of the estimation network, we propose several experiments. We use the Micro-F1 metrics ${\mathscr {M}}_{\text {mF1}}$^47^ [shown in Eq. (10)] to evaluate the accuracy of the estimation network, then we investigate the relationship between *S* and validation ${\mathscr {M}}_{\text {mF1}}$. The results in Table 1 shows that ${\mathscr {M}}_{\text {mF1}}$ of the estimation network with the standard training scheme, the DNN group, and the DCN estimator declines quickly when *S* become small, while the proposed partial label strategy can significantly reduce the required scale of training dataset. According to Table 1, we collect 64,000 randomly generated ${\mathscr {R}}$s and divide them into three datasets: 60,000 ${\mathscr {R}}$s are used as the training set, 2000 ${\mathscr {R}}$s are used as the validation set, and the rest of ${\mathscr {R}}$s are used as the testing set.

10 $$\begin{aligned} {\mathscr {M}}_{\text {mF1}} = \frac{2\sum _{c=1}^C\sum _{s=1}^S{\varvec{1}}[z_c^{\left( s\right) } = {\hat{z}}_c^{\left( s\right) }]}{\sum _{c = 1}^C\sum _{s = 1}^Sz_c^{\left( s\right) } + \sum _{c = 1}^C\sum _{s = 1}^S{\hat{z}}_c^{\left( s\right) }}. \end{aligned}$$

**Table 1:** Micro-F1 accuracy versus different number of samples.

Number of samples *S*	20,000	40,000	60,000	80,000	100,000
Estimation network with the proposed training scheme ($\%$)	76.46	85.61	89.93	90.12	89.91
Estimation network with the standard training scheme ($\%$)	52.33	61.26	74.54	82.27	89.64
DNN group^37^ ($\%$)	66.36	78.95	83.44	86.45	90.47
DCN estimator^39^ ($\%$)	62.18	71.64	80.51	85.06	89.25

We use an ULA with $M=4$ to generate these datasets according to Eqs. (4) and (5). The SNR of each sample is randomly selected in the range of $[-10\,{\mathrm {dB}}, 20\,{\mathrm {dB}}]$, and the number of snapshots *N* is fixed at 512. The training set is created as the partially labeled datasets by randomly dropping some labels per sample, while the validation set and testing set are fully labeled with the proportion of known labels of $100\%$.

The proposed estimation network is implemented with Tensorflow. The choice of network optimizer can obey the principle proposed in the prior article^44^. For PLS, if an optimizer has parameters specific learning rates (like the Adam optimizer), it is important to re-initialize the learning rates corresponding to the weights during each generation of training. For this reason, we use a common optimizer (stochastic gradient descent (SGD) optimizer) as the rule to update the weights of network. We set the learning rate of the proposed estimation network as 0.01. The hyperparameters shown in Eq. (6) are $\alpha = -4$, $\beta = 5$ and, $\gamma = 1$. According to the prior article^44,46^, we use the Bayesian uncertainty strategy with $\rho = 0.3$.

For the training generation *B* shown in the “Methods” section, we fix its value as 6 according to Table 2. Here we use the full label strategy and the standard BCE loss in the proposed estimation network. It is shown in Table 2 that $B=6$ is a cost-effective point, which has high accuracy and use less computing resource.

**Table 2:** Micro-F1 accuracy versus different number of training generation *B*.

Number of training generation *B*	3	4	5	6	8	10
Training accuracy of the proposed estimation network ($\%$)	82.07	83.75	84.63	86.33	87.06	87.24
Validation accuracy of the proposed estimation network ($\%$)	78.23	80.54	82.92	84.46	84.71	85.27

**Figure 3:**
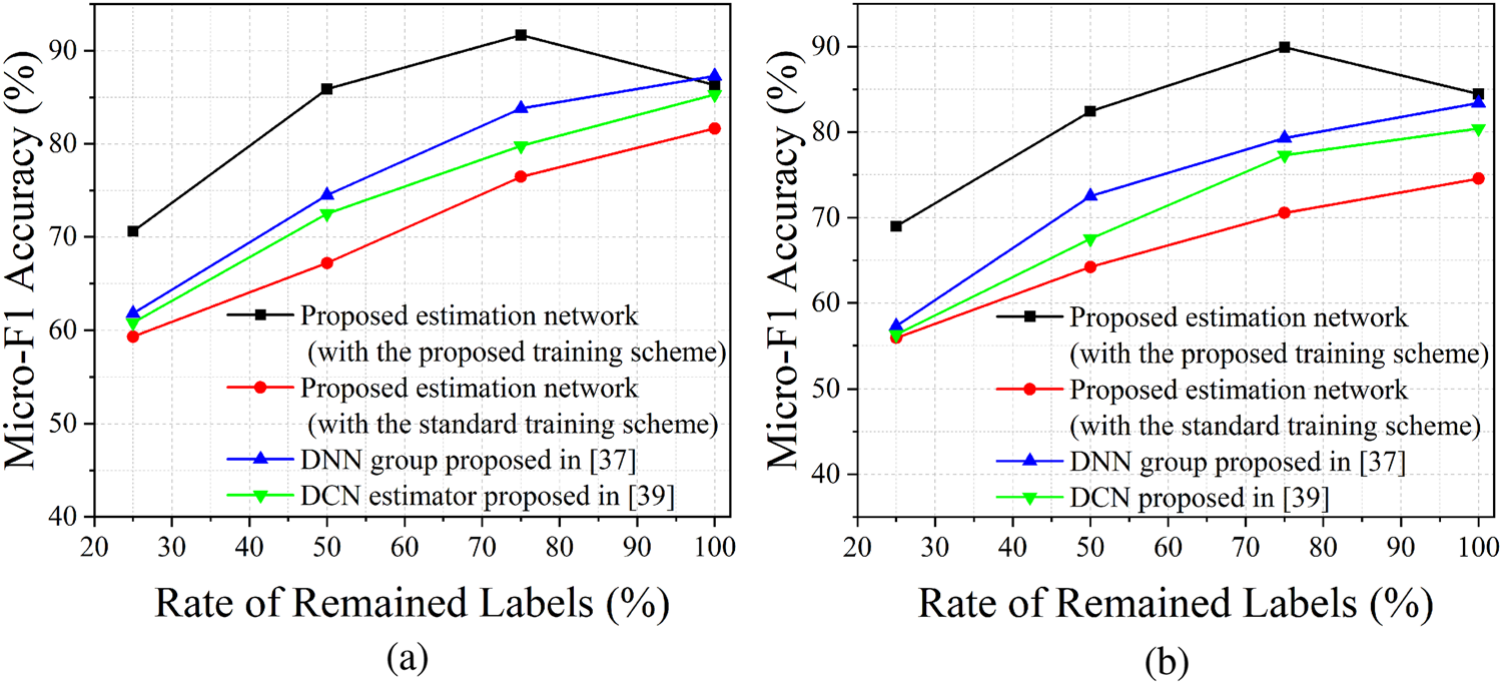
Micro-F1 accuracy comparison. (**a**) Training accuracy, (**b**) validation accuracy.

We also study the ${\mathscr {M}}_{\text {mF1}}$ score versus the rate of remained labels, and the results are shown in Fig. 3. We change the rate of remained labels from 75 to $0\%$ with the step of $25\%$ and record the accuracy performance of the proposed estimation network. It is implied that the estimation network with standard training procedure, the DNN group, and the DCN estimator cannot obtain high ${\mathscr {M}}_{\text {mF1}}$ accuracy when labels are missing, while the proposed learning strategy have a good performance even when samples missing half of labels. When $p\%=75\%$, the proposed network shows the best performance in both training and validation accuracy.

### Ablation study and training performance

We give the analysis of the importance and contribution of each proposed technology. We perform an ablation study on the training and validation sets. The results are shown in Table 3, where we set the rate of remained labels as $75\%$ in the partial-label situation. Table 3 shows that CLS and PLS can highly increase the ${\mathscr {M}}_{\text {mF1}}$ accuracy of the proposed estimation network, respectively. These two technologies can also solve the over-fitting problem. The standard BCE loss is not suitable for PLS. When partial labels collaborate with the standard BCE loss, the ${\mathscr {M}}_{\text {mF1}}$ accuracy of the proposed estimation network decreases around $7\%$. Finally, when CLS and PLS work together, the generalization of the proposed estimation network shows a surprising improvement.

**Table 3:** Ablation study of the proposed method.

CLS	Full-label	Partial-label	BCE loss	Partial-BCE loss	Micro-F1 accuracy of training set (%)	Micro-F1 accuracy of validation set (%)
	$\checkmark$		$\checkmark$		81.64	74.54
$\checkmark$	$\checkmark$		$\checkmark$		86.33	84.46
		$\checkmark$	$\checkmark$		72.47	65.81
		$\checkmark$		$\checkmark$	84.94	83.12
$\checkmark$		$\checkmark$		$\checkmark$	91.68	89.93

**Figure 4:**
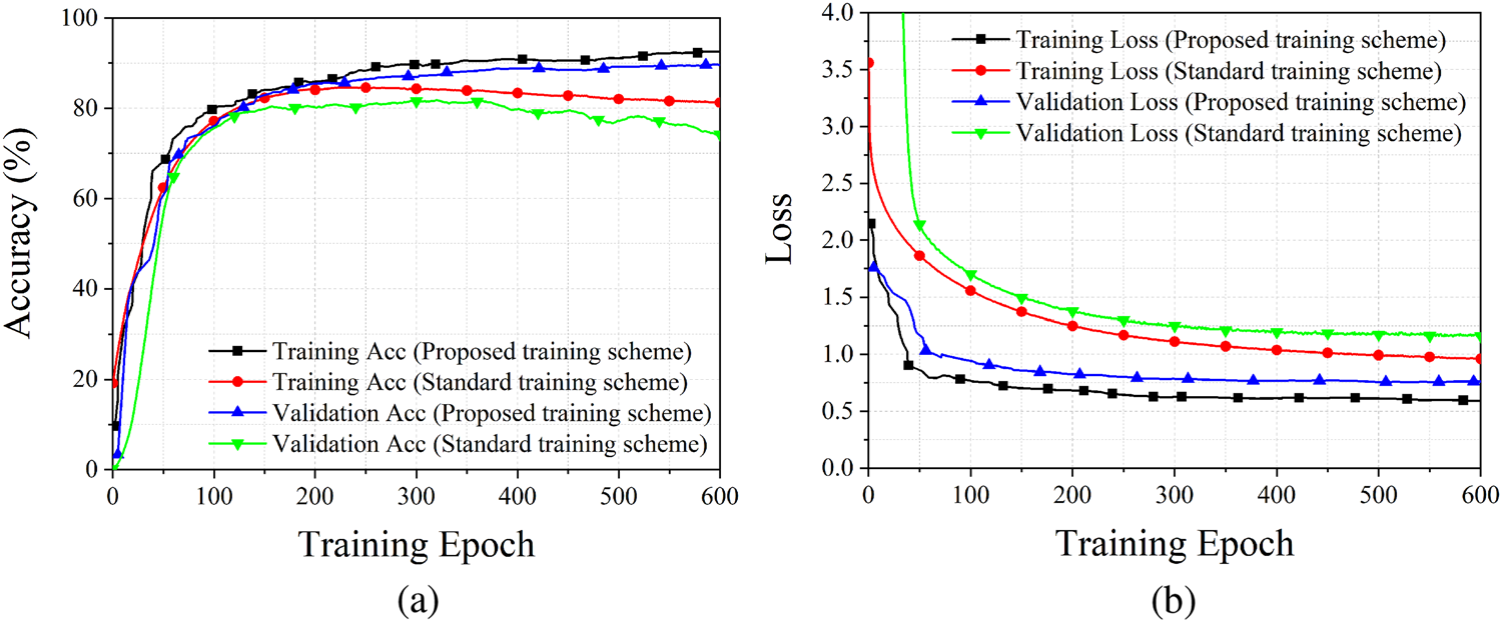
Training performance of the proposed method and standard training scheme. (**a**) Training and validation accuracy, (**b**) training and validation loss.

Figure 4 shows the training and validation performance of proposed training scheme and standard training scheme, here we fix the rate of remained labels as $75\%$ and implement 100 independent times of training for each sub-dataset. The total training time is 600. The ${\mathscr {M}}_{\text {mF1}}$ comparison results illustrate that standard training scheme can cause serious over-fitting problem, while CLS technology can overcome this problem and greatly improve the generalization of the proposed estimation network.

By using the PLS and CLS training technologies, the final ${\mathscr {M}}_{\text {mF1}}$ validation accuracy of the proposed estimation network can reach around $90\%$, which means the proposed estimation network is well trained. After training the network, we can implement some performance test corresponding to the stability of the proposed estimation network.

### Estimation performance

**Figure 5:**
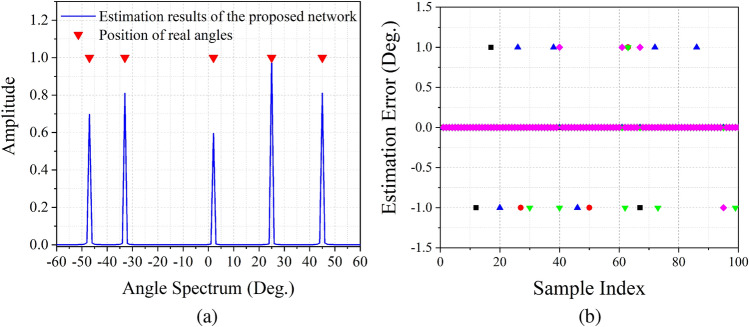
Estimation results of integer $\theta$. (**a**) Angle spectrum obtained by the proposed estimation network, (**b**) estimation error for 100 Monte Carlo experiments.

The angle spectrum obtained from the proposed estimation network is a discrete spectrum, therefore, we test the estimation performance of integer $\theta$ at first. Assuming 5 incoherent signals impinging at the direction of ${\varvec{\theta} }=\left[ -47^\circ , -33^\circ , 2^\circ , 25^\circ , 45^\circ \right]$. The SNR (resp. number of snapshots *N*) is fixed at $10\,{\mathrm {dB}}$ (resp. 512). Figure 5a shows the estimation spectrum. It is illustrated that the proposed estimation network can obtain a MUSIC-liked angle spectrum, and can estimate more signals than the number of physical sensors. We also randomly changing the SNR from $-10$ to $10\,{\mathrm {dB}}$ and implement 100 Monte Carlo experiments. The record of estimation error shown in Fig. 5b implies that DOA may drop in the neighboring grid with the probability only around $5\%$ . Both figures prove that the proposed estimation network can obtain high estimation accuracy for integer $\theta$.

**Figure 6:**
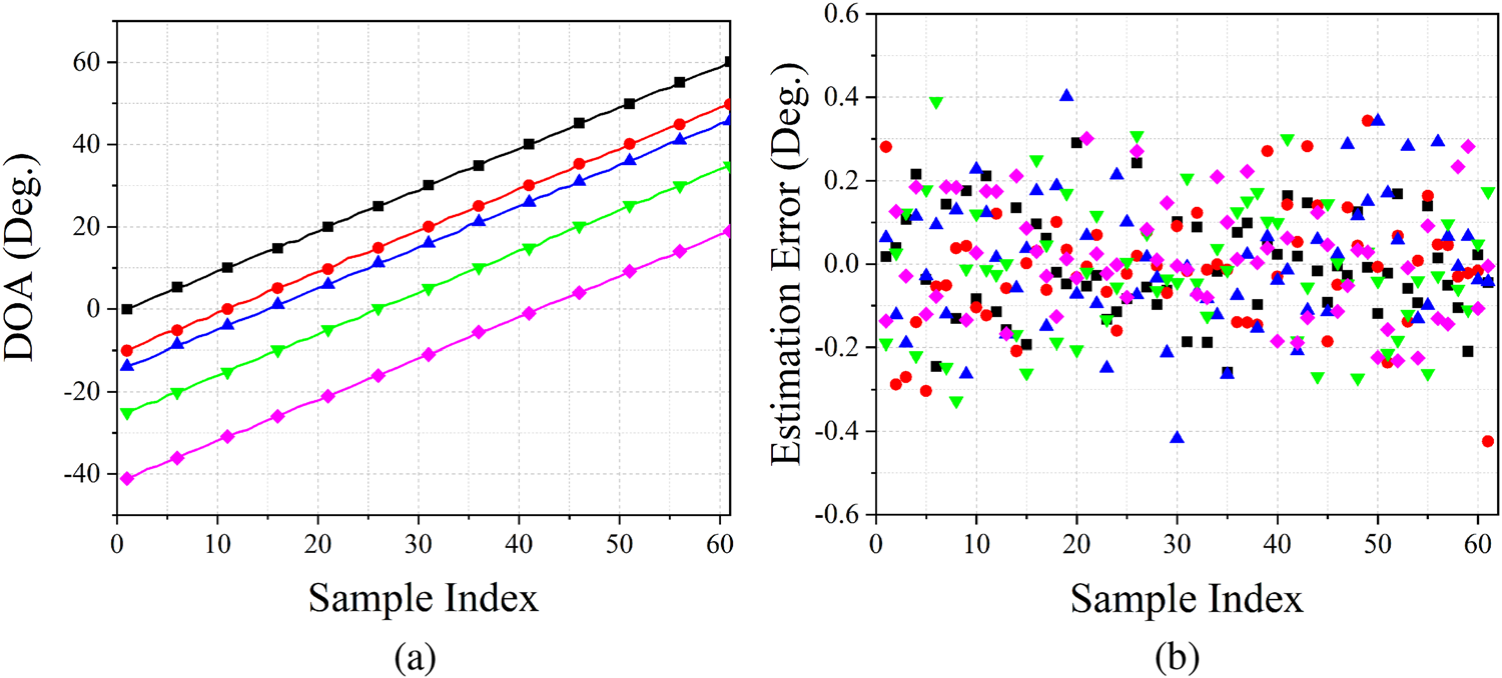
Estimation results of non-integer $\theta$. (**a**) Estimation results of each sample, (**b**) estimation error of each sample.

In order to estimate non-integer $\theta$ and achieve high-resolution results, we take the method of amplitude interpolation. The estimation of non-integer $\theta$ can be calculated as Eq. (11).

11 $$\begin{aligned} \theta _I = I + \frac{5}{4}\sum _{j = 1}^3 j(z_{I + j} - z_{I - j}), \end{aligned}$$

where *I* denotes the *I*th peak of the output spectrum. We set 5 non-integer signals to test the estimation performance of the proposed estimation network, and the estimation results (under SNR = $0\,{\mathrm {dB}}$ situation) is shown in Fig. 6. It is implied that the estimation error is fluctuating around $0^\circ$, and the maximum error is no more than $0.5^\circ$. Figure 6 also illustrates that the proposed estimation method can cover the whole range of $\theta$ and achieve a high accuracy performance for estimating non-integer $\theta$.

### Stability of the proposed estimation network

This experiment investigates the stability of the proposed estimation network. We compare the proposed method with other methods, including MUSIC algorithm^6^, RBF-NN^28^, DNN group^37^, and DCN estimator^39^. The results are obtained based on 200 times independent Monte Carlo experiments. Here we use the root mean squared error (RMSE) to evaluate the performance of all methods, which can be calculated as Eq. (12).

12 $$\begin{aligned} {\text {RMSE}} = \sqrt{\frac{1}{PK}\sum _{p=1}^P\sum _{k=1}^K \left( \theta _{kp} - {\hat{\theta }}_{kp}\right) ^2}, \end{aligned}$$

where *P* denotes the number of Monte Carlo experiments. ${\hat{\theta }}_{kp}$ (resp. $\theta _{kp}$) is the *k*th real angle (resp. estimated angle by the proposed estimation network).

Firstly, we investigate the relationship between RMSE and SNR. We fix the *N* at 512 and change the SNR from $-10$ to 20 dB. According to Fig. 6a, the RMSE response of RBF-NN method has the worst performance, the value of RMSE become lower than $1^\circ$ when SNR is higher than $2\,{\mathrm {dB}}$. The curves of DNN group and MUSIC algorithm have similar performance when SNR is higher than $0\,{\mathrm {dB}}$, while MUSIC algorithm performs better than DNN group in the case of low SNR situation. The RMSE of DCN estimator is pretty low when SNR is higher than $0\,{\mathrm {dB}}$, but the curve rises sharply as the SNR decreases. Compared with these four methods, the RMSE of proposed estimation network has the best performance, and the RMSE is still lower than $1^\circ$ when SNR $= -10\,{\mathrm {dB}}$.

We also investigate the relationship between RMSE and number of snapshots *N*. Figure 6b shows the RMSE results versus *N*, here we fix the value of SNR at 10 dB. The RMSE responses of these methods (except RBF-NN method) reveal an inverse proportional function character. For the proposed estimation network, the RMSE remains low when *N* is greater than 16. This implies that the proposed estimation network can finish the estimation task precisely by using only a few of snapshots. However, when *N* is set to be 4, the RMSE rises to around $1^\circ$, which depicts that the proposed estimation network cannot apply to single snapshot estimation processing.

**Figure 7:**
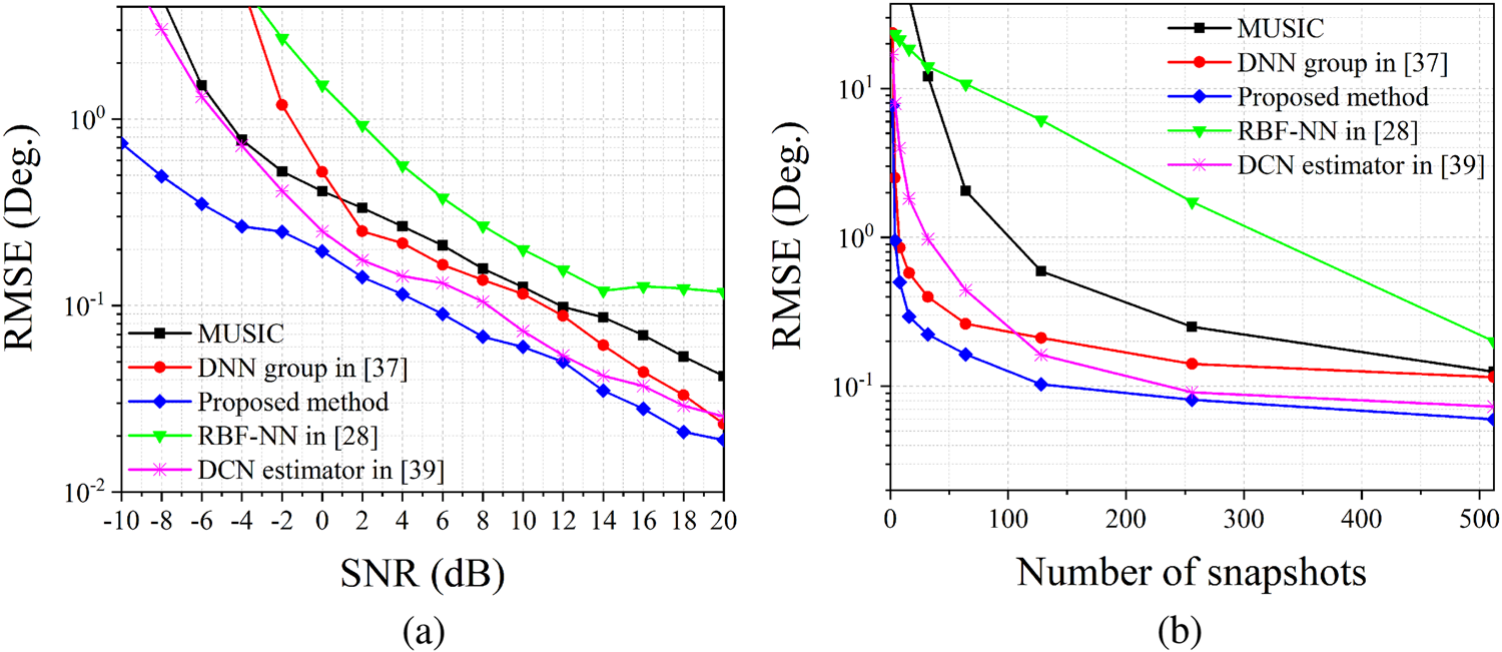
RMSE versus number of snapshots *N*, SNR equals to 10 dB.

### Resolution of the proposed estimation network

**Figure 8:**
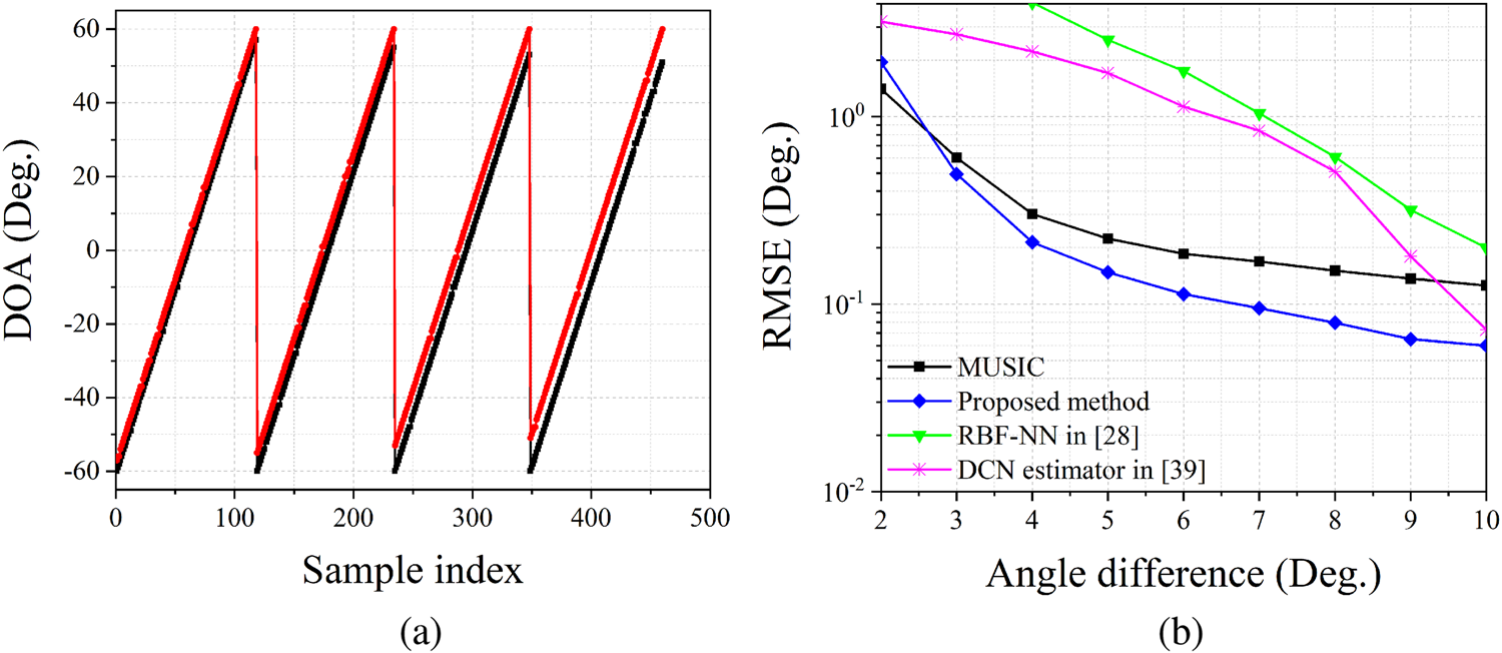
RMSE versus angle difference $\Delta \theta$, SNR equals to 10 dB and number of snapshots $N=512$.

In order to test the resolution of the proposed estimation network, we investigate the relationship between estimation performance and angle difference $\Delta \theta$. Figure 8a shows the estimation results of two $0\,{\mathrm {dB}}$ signals with a set of angular separations $\left\{ 3^\circ , 4^\circ , 5^\circ , 6^\circ \right\}$. It is obvious that the proposed estimation network can distinguish two adjacent DOAs in the sector of $[-60^\circ , 60^\circ ]$. Figure 8b shows the RMSE results versus angle difference $\Delta \theta$. Here we fix the SNR (resp. number of snapshots *N*) at $10\,{\mathrm {dB}}$ (resp. 512). The DNN group^37^ can only estimate two signals with $\Delta \theta \ge 10^\circ$, therefore, we do not show its performance in Fig. 8b. When $\Delta \theta \ge 3^\circ$, the proposed method can obtain higher estimation accuracy than other methods. When $\Delta \theta <3^\circ$ only the MUSIC algorithm and the proposed method can obtain valid estimation results. However, the precision of our proposed method is slightly lower than the MUSIC algorithm when $\Delta \theta$ become small.

## Discussion

The experiments mentioned above demonstrate the satisfying performance of the proposed method. The ablation study and training performance shown in “Results” section illustrate that CLS and PLS can improve the generalization of the proposed estimation network. The results in Figs. 3, 4, and Table 1 illustrate that CLS and PLS are suitable for solving the DOA estimation problem. These two training technologies can improve the generalization of the proposed estimation network and reduce the scale of input and output space. Figures 5 and 6 prove the validity of the proposed estimation method. By using the KR product, the DOF of array has been improved, which allows us to estimate more sources than the number of physical sensors. Figure 7 shows that our proposed method has better stability than prior estimation methods, while Fig. 8 illustrate the high-resolution performance of the proposed estimation network.

The proposed estimation network performs better than the subspace based estimation methods because it is a data-driven method, and the classification results can be hardly affected by the background information (like SNR and number of snapshots). The proposed estimation network only uses the deep features hidden in ${\mathscr {R}}$ to do the estimation task, and these features only rely on the DOA information. The inessential information can be ignored by the proposed estimation network while subspace based methods and machine learning based methods can be influenced by these information.

In conclusion, an improved estimation network used for multi-DOA estimation is proposed in this paper. We discuss the image feature of tensor ${\mathscr {R}}$ obtained by the KR product. Based on the conceptions proposed in Method section, we use an efficient training scheme for the proposed estimation network. By adding the CLS and PLS into the network, the generalization of the estimation network has been greatly improved. The simulation results of all experiments show that our proposed estimation method has high estimation performance and can tolerate the harsh estimation environment.
